# An Association Study between Longitudinal Changes of Leukocyte Telomere and the Risk of Azoospermia in a Population of Iranian Infertile Men

**DOI:** 10.22034/ibj.22.4.231

**Published:** 2018-07

**Authors:** Hamed Heidary, Farkhondeh Pouresmaeili, Reza Mirfakhraie, Mir Davood Omrani, Hamid Ghaedi, Zahra Fazeli, Shadi Sayban, Soudeh Ghafouri-Fard, Eznollah Azargashb, Fazlollah Shokri

**Affiliations:** 1 Department of Medical Genetics, Faculty of Medicine, Shahid Beheshti University of Medical Sciences, Tehran, Iran; 2 Infertility and Reproductive Health Research Center (IRHRC), Shahid Beheshti University of Medical Sciences, Tehran, Iran; 3 Department of Community Medicine, Faculty of Medicine, Shahid Beheshti University of Medical Sciences, Tehran, Iran

**Keywords:** Azoospermia, Male infertility, Spermatogenesis, Telomere

## Abstract

**Background:**

Telomeres are evolutionary, specialized terminal structures at the ends of eukaryotic chromosomes containing TTAGGG repeats in human. Several human diseases have been known to be associated with dramatic changes in telomere length. The aim of the present study was to assess the correlation between the relative leukocyte telomere length (LTL) and infertility in a group of Iranian azoospermic males.

**Methods:**

In this case-control pilot study, relative telomere length (RTL) of peripheral blood leukocytes from a total of 30 idiopathic non-obstructive azoospermic males and 30 healthy fertile males was evaluated using real-time PCR. RTL was calculated as T (telomere)/S (single copy gene) ratio and compared between infertile and fertile groups.

**Results:**

Patients with azoospermia showed significantly shorter RTL than fertile males (0.54 vs. 0.84, *p* < 0.05). The area under the receiver operating characteristic (ROC) curve was estimated to be 99.8%, suggesting LTL as a potential marker for the diagnosis of azoospermia.

**Conclusion:**

Our findings demonstrated a probable association between telomere shortening and azoospermia in a population of Iranian infertile men affected by idiopathic azoospermia.

## INTRODUCTION

In general, infertility is defined as failure in being pregnant after one year of regular unprotected intercourse. Infertility affects almost 15% of couples[1,2]. Among known causes for infertility, male-related factors contribute to the almost 50% of the cases[3,4]. Azoospermia is one of the causes of infertility in males that can be defined as the absence of sperm in semen. This condition is observed in about 1% of all men in a general population and in 10-15% of all infertile males[4]. There are two different clinical types of azoospermia: obstructive azoospermia (OA) and non-obstructive azoospermia (NOA). OA affects 40% of azoospermic men and arises secondary to the obstruction of the male reproductive tract[5]. Patients affected by OA are evaluated by patient’s history, physical examination, CFTR mutation screening, and imaging studies. NOA consists of primary and secondary testicular failure and accounts for 60% of azoospermia cases. There are various approaches to distinguish between OA and NOA, including clinical evaluation, hormone assessment, and chromosomal and genetic tests for determination of karyotype, Y chromosome microdeletion, or hypogonadotropic hypogonadism[6,7]. Although numerous studies have performed in azoospermia, few reports have been emerged in the association of infertility and telomere length[8].

Telomeres are known to be evolutionary, specialized DNA microsatellites located at the end of linear chromosomes. They are composed of TTAGGG repeats in humans and associated with specific proteins such as shelterin and telomerase[9]. Telomeres play an important role in the protection of chromosome ends from degradation and also in chromosome fusion[10]. DNA polymerase does not have ability to complete the replication of linear chromosome ends, but telomerase are able to solve this problem by synthesis of telomeres and can prevent the loss of important genetic information during DNA replication[11,12]. Telomere length varies greatly among different species, from approximately 300 bp in yeast to many kilobases in human and mouse[13]. Previously, it has been indicated that females have longer telomeres than males[14]. The shortening of telomere induces senescence in cells and prevents the cancer[15]. Investigations have demonstrated that telomere shortening through cell divisions leads to early cell senescence and is associated with the etiology of some diseases, including idiopathic pulmonary fibrosis, cardiovascular diseases, ulcerative colitis, diabetes mellitus, Alzheimer’s and Parkinson’s diseases, gastric carcinoma, breast cancer, acquired immunodeficiency syndrome (AIDS), and male infertility[8,16-25]. Moreover, telomere shortening has been indicated to play a major role in aging. It has been proposed that telomere attrition can induce apoptosis and cell cycle arrest, thereby leading to cell loss and tissue dysfunction[26].

Sperm telomere length (SLT) has an association with sperm count and chromosome abnormality, and it is lower in oligozoospermic than normozoospermic semen. Furthermore, shorter telomeres have been observed in infertile than fertile males[27]. Currently, a survey has demonstrated that SLT is connected with the motility and vitality of sperm, as well as sperm DNA fragmentation[28]. Another investigation on adult male Brown Norway rats has indicated an association between chemotherapy treatment and the reduction of the telomere length in spermatocytes[29]. Another study has found that the exposure to anticancer drugs may have a function in male infertility through the induction of telomere dysfunction[30]. Although there are numerous reports about relationship between male infertility and STL[8,31,32], the correlation between azoospermia and leukocyte telomere length (LTL) has not been examined yet. A previous study has shown that the LTL is significantly correlated with SLT[27]. In the present study, our aim was to elucidate the possible genetic association between the relative telomere length (RTL) of leukocytes and male infertility in Iranian males affected by azoospermia.

## MATERIALS AND METHODS

### Azoospermia patients and controls

The present study was performed on 30 non-obstructive azoospermic patients with a normal karyotype who were screened for the presence of Y chromosome microdeletion. The patients were candidates for intracytoplasmic sperm injection and referred to the National Institute of Genetic Engineering and Biotechnology (NIGEB; Tehran, Iran). Semen analysis was performed according to WHO normal standard parameters. Urological examination was conducted in all the patients for anatomical integrity of genital system. Hormone analysis, including follicle-stimulating hormone (FSH) and luteinizing hormone (LH) were also done. The patients had normal chromosomal complement and intact Y chromosome; these data were extracted from the medical history of the patients. The patient group was matched to the control group consisting of 30 fertile males, regarding their lifestyle and age. The average age of the patients and controls were 35.4 years (standard deviation [SD] = 4.52). The clinical evaluation of the patients indicated no signs of cryptorchidism, varicocele, hypogonadism, and hypospadias. Informed consents were obtained from all individuals participated in the study. This work was approved by the Ethics Committee of Shahid Beheshti University of Medical sciences, Tehran, Iran.

### DNA isolation and real-time PCR analysis

Genomic DNA was extracted from peripheral blood samples by Diatom kit (GenFanAvaran, Tehran, Iran). The samples were then quantified by using a Nano Drop (Biochrome Ltd., UK) and by electrophoresis on 2% agarose gel. Telomere length of blood leukocytes was determined by a quantitative real-time PCR-based method as previously described[33]. Briefly, the average ratio of telomere repeat copy number (T) to the single gene copy number (S) was determined. The measurement of T and S was performed in two separate quantitative PCR reactions of 20 μl total volume containing 30 ng of DNA, 10 µl of 2× real-time PCR Master Mix SYBR Green without ROX (Pishgam Biotech Co., Tehran, Iran), and the suitable concentration of each primer (270 nM primer Tel1 and 900 nM primer Tel2 or 300 nM of each hemoglobin gamma gene (HBG) 1 and HBG2). The oligonucleotide primers were as follows: Tel1F: 5’GGTTTTTGAGGGTGAGGGTGAGGGTGAGGGTGAGGGT3’; Tel2R: 5’TCCCGACTATCCCTATCC CTATCCCTATCCCTATCCCTA3’; HBG1F: 5’GCT TCTGACACAACTGTGTTCACTAGC3’ and HBG2R: 5’CACCAACTTCATCCACGTTCACC3’.

The HBG was used to normalize the telomere quantitative PCR signal as previously demonstrated[33]. All the samples were run in triplicate, and all quantitative PCR reactions were performed in a Rotor-Gene 6000 system (Corbett Research Ltd., Cambridge, UK). The PCR amplification consisted of an initial denaturation at 95 °C for 10 min, followed by 22 and 25 cycles of denaturation at 95 °C for 15s and annealing/extension at 54 °C for 2 min or 58 °C for 1 min to determinate T and S, respectively. The specificity of PCR products was confirmed by drawing melting curves and electrophoresis on 2% agarose gel. The efficiency range of PCR reactions was 1.7 to 1.9 (85-95%). The T/S ratio was calculated using the following formula: [2^C^_t_^(telomeres)^/2*^C^*_t_(*^HBG2)]–1^ = 2^–Δ*C*^_t_*.

### Statistical analysis

Normality test was performed by the Kolmogorov-Smirnov test. Quantitative variables were expressed as mean ± SD or median (interquartile range), depending on whether the data were normally distributed. Student’s *t*-test was used to evaluate T/S ratio between males affected by azoospermia and fertile males. All the statistical analysis was performed using SPSS version 22.0. *p* value less than 0.05 was considered statistically significant. The application of telomere length, as a diagnostic marker of azoospermia, was evaluated using the receiver operating characteristic (ROC) curve analysis.

## RESULTS

**Fig. 1:**
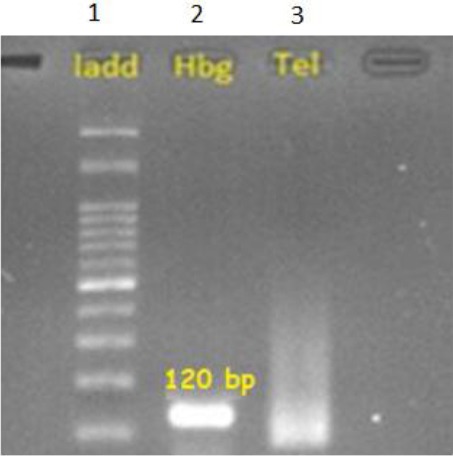
Agarose gel electrophoresis of the PCR products obtained for the analysis of telomere length. Lane 1, DNA ladder; lane 2, amplification of HBG producing a 120-bp fragment; lane 3, amplification of telomere region yielding a smear

The mean values of FSH and LH serum levels were 35.50 mIU/ml (Normal FSH 2-7 mIU/ml). and 13.69 mIU/ml in patients (Normal LH 0.7 to 7.9 IU/l), respectively. LH and FSH can be low or elevated in NOA[34]. The PCR products for telomere region yielded a smear with high intensity at less than 100 bp and the low density at larger size (Fig. 1). The product of HBG amplification was 120 bp as expected. We compared RTL in patients with azoospermia to those of control group by quantitative PCR.

**Fig. 2:**
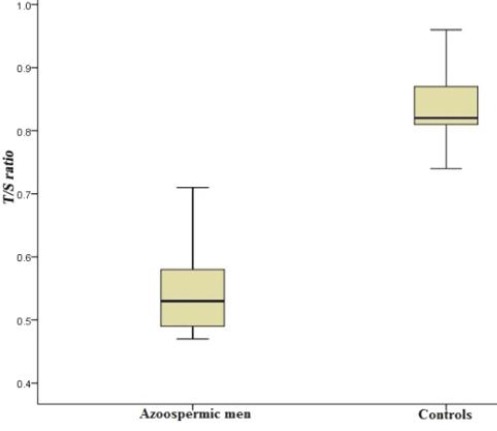
The distribution curve of T/S (relative telomere length) in patients (males affected by azoospermia) and controls (fertile males). The vertical lines show the minimum and maximum values. The relative leukocyte telomere length to HBG (T/S) ratio of males affected by azoospermia was significantly lower (*p* < 0.05, 95% CI) than fertile males (0.54 vs. 0.84). The lowest value of this ratio was 0.47 and 0.74, and the highest level identified to be 0.71 and 0.96 in azoospermic males and fertile males, respectively.

**Fig. 3:**
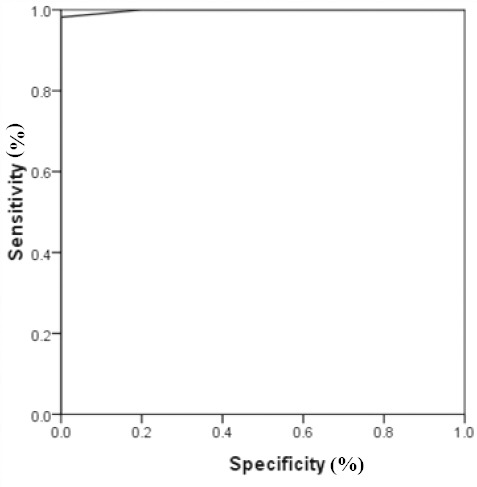
ROC curve plotted for leukocyte telomere length as a diagnostic marker of azoospermia using T/S values (area under the ROC curve = 99.8%).

The results from statistical analysis indicated that the relative LTL to HBG (T/S) ratio of males affected by azoospermia was significantly lower (*p* < 0.05, 95% CI) than fertile males (0.54 vs. 0.84). The lowest value of this ratio was 0.47 and 0.74, and the highest levels were identified to be 0.71 and 0.96 in azoospermic males and fertile males, respectively (Fig. 2). Also, the mean of ΔCt in the males affected by azoospermia was observed to be significantly higher (*p* < 0.05, 95% CI) than the fertile males (7.9 vs. 2.69). The lowest amounts of ΔCt were 6.3 and 0.5, and the highest levels of this value were reported to be 9.3 and 4.5 in azoospermic and fertile males, respectively. The amplification was performed with the efficiency of the range of 1.7-1.9. Furthermore, the ROC curve analysis suggested that LTL could be considered as a strong marker for the presence of azoospermia (Fig. 3; area under the curve 99.8%).

## DISCUSSION

Telomere length has been recognized to have an important function in the maintenance of genome integrity, recombination, and meiosis division[35]. Different diseases have been reported to be connected with telomere shortening[36], but the precise role of telomere length in male fertility remains to be investigated. A previous study on males affected by azoospermia has indicated that the shorter telomere length is found in the sperms of azoospermic males than fertile males, suggesting a possible contribution of telomere length in some unexplained male infertilities[8]. Our results showed that there was a promising association between telomere length and the occurrence of azoospermia even if the telomere size was evaluated in the blood leukocytes of azoospermic males. In a study by Ferlin *et al*.[27] it has been found that LTL was significantly correlated with SLT. Moreover, STL is related to sperm count. However, the relationship between LTL and sperm count did not demonstrate a significant correlation[27].

Considering the role of telomeres in meiosis and maintenance of genome integrity, the telomere shortening might lead to impaired spermatogenesis, followed by germ cells death. In fact, the increased expression of telomerase in testis in primary spermatocytes is consistent with its role in meiosis I[37].

Many factors have been known to be involved in male infertility through spermatogenic impairment. Some of them include environmental factors, infections, oxidative stress, smoking, and obesity that may affect spermatogenesis by telomere shortening or its regulation[38]. To better understand the effect of these factors on longitudinal telomeric changes in sperm, peripheral blood sampling can be a good candidate to avoid testicular biopsy. Average LTL is just a biological marker, which could be affected by several factors such as ethnicity, gender, age, lifestyle, health status (including diseases like coronary artery diseases) and even environmental factors. The average LTL in humans has been reported to be associated with telomere length in other tissues[39]. Therefore, while telomere size in leukocytes does not demonstrate the exact molecular mechanism of azoospermia, it seems that the genetic variation is appropriate for a more in-depth study of the role of telomeres in male infertility.

Our results suggest a relationship between shorter telomeres and the increased risk of azoospermia. Moreover, this pilot study showed that the RTL in blood can be considered as a potential new biological indicator of either some azoospermic cases or unknown genomic events, leading to spermatogenesis in the testis. Further studies are needed to confirm our findings in a larger population.
